# Supplementary material for: Exposure to House Dust Mite Allergen and Endotoxin in Early Life and Sensitization and Allergic Rhinitis: The JECS
Source: Int J Environ Res Public Health. 2022 Nov 10;19(22):14796. doi: 10.3390/ijerph192214796 (PMC9690251; doi:10.3390/ijerph192214796)
Supplement: Supplementary file 1 [file ijerph-19-14796-s001.zip › ijerph-1999328-supplementary.pdf]

Table S1. Log-transformed IgE to house dust mite allergen in relation to endotoxin or Der 1 exposure in dust (multivariate linear regression analysis).

	Der f 1 sensitization			Der p 1 sensitization		
	Coefficient	SE	P value	Coefficient	SE	P value
Endotoxin (EU/m^2, log$_{10}$ transformed)	**0.49**	**0.05**	**< 0.01**	**0.72**	**0.06**	**< 0.01**
Der 1 (ng/m^2, log$_{10}$ transformed)	**0.14**	**0.04**	**< 0.01**	0.07	0.05	0.12

SE, standard error. Model was adjusted for parental history of allergy, passive smoking, house income, mode of delivery, birth weight, child sex, older siblings, exclusive breast feeding, daycare attendance at one year, and household pet. Boldface indicates statistical significance ($p < 0.05$).

Table S2. Odds ratios of sensitization to Der1 in relation to endotoxin or Der 1 exposure in the season status subgroup.

		Der f 1 sensitization			Der p 1 sensitization		
		aOR	95%CI		aOR	95%CI	
Fall and winter (October to March)							
Endotoxin (EU/m²)							
Q1	< 186.9	ref			ref		
Q2	≥ 186.9, < 375.1	1.25	0.79,	1.99	1.26	0.79,	2.00
Q3	≥ 375.1, < 826.5	1.34	0.86,	2.11	1.26	0.80,	1.99
Q4	≥ 826.5	**1.61**	**1.04,**	**2.50**	**1.74**	**1.12,**	**2.70**
Der 1 (ng/m²)							
Q1	< 14.8	ref			ref		
Q2	≥ 14.8, < 51.2	1.00	0.66,	1.54	1.14	0.75,	1.76
Q3	≥ 51.2, < 168.6	**1.69**	**1.14,**	**2.52**	**1.86**	**1.24,**	**2.79**
Q4	≥ 168.6	**2.88**	**1.93,**	**4.29**	**2.99**	**1.99,**	**4.50**
Spring and summer (April to September)							
Endotoxin (EU/m²)							
Q1	< 186.9	ref			ref		
Q2	≥ 186.9, < 375.1	1.13	0.70,	1.83	1.25	0.77,	2.01
Q3	≥ 375.1, < 826.5	1.06	0.63,	1.78	1.09	0.64,	1.86
Q4	≥ 826.5	1.32	0.73,	2.36	1.28	0.70,	2.33
Der 1 (ng/m²)							
Q1	< 14.8	ref			ref		
Q2	≥ 14.8, < 51.2	1.28	0.65,	2.53	1.01	0.51,	2.00
Q3	≥ 51.2, < 168.6	1.70	0.88,	3.29	1.79	0.95,	3.38
Q4	≥ 168.6	**3.45**	**1.79,**	**6.62**	**2.76**	**1.46,**	**5.24**

Abbreviations: CI, confidence interval; OR, odds ratio. Model 1: crude; Model 2: Adjusted for parental allergy history, passive smoking, household income, mode of delivery, birth weight, child's sex, older siblings, exclusive breastfeeding, daycare attendance at 1 year, and household pet. Boldface indicates statistical significance ($p < 0.05$).
